# Vitamin D Significantly Inhibits Carcinogenesis in the Mogp-TAg Mouse Model of Fallopian Tube Ovarian Cancer

**DOI:** 10.3390/nu16193318

**Published:** 2024-09-30

**Authors:** Omar L. Nelson, Rebecca Rosales, Jane Turbov, Larry G Thaete, Gayathriy Balamayooran, J Mark Cline, J. Wesley Pike, Gustavo C. Rodriguez

**Affiliations:** 1 Department of Obstetrics and Gynecology, Division of Gynecologic Oncology, Endeavor Health, Evanston, IL 60201, USA; onelson@northshore.org (O.L.N.); rrosales@northshore.org (R.R.); jturbov@northshore.org (J.T.); lthaete@sbcglobal.net (L.G.T.); 2 Department of Obstetrics and Gynecology, University of Chicago Pritzker School of Medicine, Chicago, IL 60637, USA; 3 Pathology/Comparative Medicine and Radiation Oncology, Wake Forest School of Medicine, Winston-Salem, NC 27157, USA; gabalama@wakehealth.edu (G.B.); jmcline@wakehealth.edu (J.M.C.); 4 Department of Biochemistry, University of Wisconsin-Madison, Madison, WI 53706, USA; jpike@wisc.edu

**Keywords:** vitamin D, cholecalciferol, chemoprevention, carcinogenesis, mogp-TAg, fallopian tube epithelium

## Abstract

Epidemiological and observational studies suggest that vitamin D has potential for the chemoprevention of ovarian cancer. The anticancer effect of vitamin D in the fallopian tube epithelium (FTE), which is now thought to harbor the precursor cells for high grade ovarian cancer, is not known. The purpose of this study was to investigate whether vitamin D can inhibit carcinogenesis in the mogp-TAg fallopian tube (FT) ovarian cancer mouse model and examine underlying mechanisms. To test this hypothesis, 3 groups of 40 5-week-old female mogp-TAg mice were divided equally into two cohorts of 20 mice, treated with either vehicle (vitamin D solvent) or the active 1,25(OH)2D3 analogue EB1089, delivered via mini-pump or IP injection or cholecalciferol delivered in the feed. The FTs were characterized histologically and pathologically after 3 and 7 weeks of treatment. The effect of vitamin D on cultured human FTE cells was also examined. After 3 weeks, vitamin D, delivered as either cholecalciferol or EB1089 significantly inhibited FT carcinogenesis. After 7 weeks, cholecalciferol significantly reduced p53 signatures, serous tubal epithelial carcinoma, FT cancer, and plasma CA125 while increasing apoptosis in the FTE. EB1089 had no significant effect on FT carcinogenesis at 7 weeks. Cholecalciferol significantly reduced proliferation and increased apoptosis in vitro in p53-altered FTE cells. In conclusion, vitamin D inhibited FT carcinogenesis by clearing cells with p53 alterations. These data suggest that vitamin D has merit for the chemoprevention of fallopian tube/ovarian cancer. The optimal chemopreventive effect may be dependent on the route of vitamin D administration

## 1. Introduction

Epithelial ovarian cancer (EOC) remains a significant public health problem. It is the sixth-leading cause of cancer-related mortality in women in the United States, causing approximately 40% of deaths due to gynecologic malignancy [1]. As a consequence of the nonspecific subtle symptoms associated with early-stage ovarian cancers, along with a lack of an effective screening test, most women have advanced-stage malignancy. For these women, overall survival remains poor (less than 30%) [2]. Thus, there is a dire need for development of better methods of prevention, early detection, and treatment to eradicate this devastating disease.

In recent years, there has been a paradigm shift in our understanding of the early events underlying ovarian carcinogenesis. It is becoming more accepted that the epithelium in the distal fimbriated end of the fallopian tube (FT) harbors the cell of origin for many high-grade epithelial ovarian cancers. Intrinsic and extrinsic genotoxic factors lead to the accumulation of mutated p53 protein in secretory cells in the fallopian tube epithelium (FTE), leading to the p53 signature. Subsequent acquisition of secondary genomic alterations ultimately leads to the development of serous tubal intraepithelial carcinoma (STIC). STIC lesions can further evolve into invasive carcinoma, which spreads via shedding/exfoliation directly to the adjacent ovary and into the abdominal cavity [3,4]. The discovery that these changes in the FTE represent the earliest steps in ovarian carcinogenesis opens the door to the development of pharmacologic strategies that can arrest and/or reverse the early transformative events in the fimbria, with the potential to decrease ovarian cancer incidence and mortality through prevention.

Ecological studies dating back to the 1980s have shown a geographic distribution of a number of cancers including ovarian cancer. Incidence and/or mortality are inversely correlated with exposure to sunlight, with increasing cancer risk associated with greater distance from the equator, north, or south. Since the primary source of vitamin D is via endogenous production in the skin exposed to UVB radiation, it was hypothesized that vitamin D might have a chemopreventive effect on cancer [5,6,7,8,9].

Cholecalciferol (Chole), which is metabolically inactive and non-toxic, is obtained primarily via endogenous production in the skin but also via the diet. It is hydroxylated in the liver to 25(OH)D3 and further metabolized in the kidney and at the tissue level in many types of epithelia via 25-hydroxyvitamin D3 1-alpha-hydroxylase (CYP27B1) to the active hormone 1,25(OH)2D3 (calcitriol) (CAL). Calcitriol then interacts with the vitamin D receptor (VDR), which is expressed ubiquitously throughout most epithelia. Vitamin D-responsive genes have been shown to confer a number of biologic effects that have the potential to arrest or reverse carcinogenesis. Of note, via expression of the 1-alpha hydroxylase enzyme, many normal tissues, including those from the gynecologic tract, have the capacity to convert circulating 25(OH)D3 to 1,25(OH)2D3, which can act in an autocrine or paracrine fashion to regulate cell growth and biology [9,10,11,12].

The murine oviduct-specific glycoprotein promotor-driven simian virus 40 large T-Antigen (mogp-TAg) transgenic mouse model of fallopian tube cancer recapitulates the cellular and molecular changes typical of human ovarian and fallopian tube cancers [13,14].

Similar to humans, the inactivation of p53 is an early event in fallopian tube carcinogenesis in this mouse model, with subsequent development of p53 signatures, STICs, and cancer. Using this mouse model, we have previously shown that the candidate ovarian cancer preventive progestin not only markedly inhibited the development of fallopian tube cancer but also eradicated histologically normal-appearing FTE cells with abnormal p53, the genetic alteration thought to represent the earliest putative event in fallopian tube carcinogenesis [15]. Identification of drugs that have a similar biological effect in the FTE may open the door to the discovery of other agents with the potential to significantly prevent ovarian cancer.

Little is known about the effect of vitamin D on FT carcinogenesis. The goal of this study was to directly test the cancer-preventive effects of vitamin D in the fallopian tube. In addition, we sought to characterize the biological mechanisms underlying the chemopreventive effect of vitamin D in the fallopian tube.

## 2. Materials and Methods

### 2.1. Transgenic Mice

Female mogp-TAg mice were derived as described previously; a 2.2 kb segment of the 5′-flanking sequence of the mouse oviduct-specific glycoprotein (OGP) gene is used to drive expression of the simian virus 40 large T-Antigen (SV40-TAg), leading to development of tumors in the gynecologic tract [13]. Female mogp-TAg mice were obtained from The Jackson Laboratory (Bar Harbor, ME, USA) at 5 weeks of age. The mice were then housed at the Endeavor Health Center for Comparative Medicine with ad libitum access to a phytoestrogen-free diet and to water in 12 h light/dark cycles. The weight of the mice was measured weekly from the start to end of each trial.

### 2.2. Trial Design

Under an approved IACUC protocol, 3 groups of 40, 5-week-old female mogp-TAg mice were each divided equally into two cohorts of 20 mice, treated with either vehicle (vitamin D solvent) or vitamin D (summarized below), with 20 euthanized at 8 and the other 20 at 12 weeks of age. At the end of the trial, the reproductive tracts and blood were collected from each animal. Vitamin D delivery/formulation varied across the three groups:

Trial 1—ALZET Osmotic Pump: Under isoflurane anesthesia, the mogp-TAg mice were subcutaneously implanted with an ALZET Osmotic Pump (model 1004, ALZET, Cupertino, CA, USA) through an incision between the scapulae for the delivery of vehicle (sterile polypropylene glycol and 0.05M Na_2_HPO_4_ (80:20 *v*/*v*)) or a stock solution of 100 µg/mL EB1089 (activated vitamin D analogue) (Cat #3993 Tocris, Bristol, UK) diluted so that the pumps delivered 0.15 µg/kg/day of EB1089. The pumps were replaced every 28 days.

Trial 2—Intraperitoneal (IP) Injection: Mice were injected twice per week with vehicle (sterile polypropylene glycol and 0.05M Na_2_HPO_4_ (80:20 *v*/*v*)) or 0.5 µg/kg EB1089 dissolved in sterile polypropylene glycol and 0.05M Na_2_HPO_4_ (80:20 *v*/*v*)).

Trial 3—Diet/feed: Mice were provided irradiated 14% Protein Rodent Maintenance Diet (0.72% Ca), 2914 (Envigo, Indianapolis, IN, USA), or this diet supplemented with 25 IU vitamin D (cholecalciferol) per gram of diet ad libitum.

### 2.3. Fallopian Tube Morphologic Analysis

For morphological analyses, formalin-fixed reproductive tracts of 8- and 12-week-old vehicle and vitamin D-treated mice were paraffin-embedded, sectioned at 5 µm, and then H&E stained. The reproductive tract tissues were examined histologically in a blinded fashion by two veterinary pathologists (authors Drs. J Mark Cline and Gayathriy Balamayooran) for evidence of neoplasia or other abnormalities. The histology in the fallopian tube of each mouse was ranked as follows: 0 for normal, 1 for hyperplasia, 2 for hyperplasia with atypia, and 3 for invasive carcinoma. H&E sections were microscopically examined on an Olympus BX43 at 10–40× magnification. The percentage lesion type was calculated by dividing the total number of animals with a specific lesion type by the total number of animals in the treatment group. For histologic analyses, since there were no differences in the lesion distribution in the vehicle groups across the three cohorts, we combined the vehicle animals for each trial at 8 and 12 weeks, respectively.

As previously described [15], Fiji (ImageJ 2.90) was utilized to measure the fallopian tube (FT) cross-sectional area (pixel per unit area) of H&E section images captured on an Olympus BX43 light microscope at 4× magnification in 8- and 12-week-old mice by free-hand drawing a border around each cross-section.

### 2.4. Immunohistochemistry (IHC)

IHC staining on 5 µm fallopian tube sections was accomplished by using the University of Chicago Human Tissue Resource Center and the Endeavor Health Histology Core. For IHC staining, standard protocols were used for p53 (Novacastra NCL-2-p53-CMp, 1:400, Leica Biosystems, Chicago, IL, USA), PAX8 (Proteintech, Rosemont, IL, USA, 10336-1-AP, 1:300), cleaved caspase-3 (Asp175) (5A1E) Rabbit mAb (Cell Signaling, Danvers, MA, USA, #9664, 1:200), and Ki67 (Cell Signaling D3B5 12202S, 1:200). IHC images were captured on an Olympus BX43 light microscope at 2–10× magnification using MMI cellScan.

### 2.5. Lesion Quantification

Sections from the vehicle and vitamin D-treated groups (*n* = 10 mice per group; 20 total for each time point) were examined in a blinded fashion; the total number of p53 signature and STIC lesions were counted microscopically at 4× using p53 IHC-stained sections. Also, 10× and 20× magnifications were used to confirm a defined p53 signature and STIC lesion. The total number of lesions was divided by the total number of animals to generate the average number of lesions in vehicle- and vitamin D-treated mice. In order to obtain the average number of p53 signatures and STIC lesions per cross-section, the average number of lesions was divided by the average number of cross-sections per treatment group. This calculation resulted in the average number of lesions per FT cross-section per treatment group. The sections used for p53 signature and STIC lesion quantification were within 10–15 µm of the H&E sections used for measuring the FT cross-sectional area in the vehicle and vitamin D trial groups.

### 2.6. Cleaved Caspase-3 Quantification

In a blinded fashion, an Olympus BX43 light microscope was used to count the number of cleaved caspase-3 positive cells from IHC sections at 20×. The average number of caspase-3 positive cells per FT cross-section was calculated. Cleaved caspase-3-stained sections were within 10–15 µm of the p53-stained FT sections in the vehicle and vitamin D feed group.

### 2.7. Calcium Measurements

Mouse plasma calcium concentrations at each treatment time point were determined using a calcium colorimetric assay (Sigma, MAK022-1KT, Saint Louis, MO, USA) according to the manufacturer’s instructions. A linear standard curve was generated by plotting reference standards (0–20 mg/dL) against absorbance values and calculating each specimen accordingly.

### 2.8. 25(OH) D3 Measurements in the Feed Trial

Mouse plasma (25 µL plasma/well) from the feed trial was utilized to measure 25(OH) D3 concentrations using the Mouse/Rat 25(OH) D3 ELISA kit (Eagle Biosciences, Amherst, NH, USA, Cat #VID21-KO1) according to manufacturer’s instructions.

### 2.9. Mouse Tissue and Western Blot

Kidney and FT tissue were homogenized in RIPA lysis buffer (ThermoFisher, Waltham, MA, USA, Cat #89901) with protease inhibitor cocktail (Millipore Sigma Cat #118266170001) and incubated on a shaker for 2 h in a cold room. The homogenate was centrifuged at 13.2× *g* for 30 min at 4 degrees. Then, 30 μg of protein was used for standard western blotting analysis for p53 (Novacastra NCL-2-p53-CMp, 1:1000), and vitamin D receptor (VDR-Cell Signaling, #12550S) and PAX8 (Proteintech Rosemont, IL, USA 10336-1-AP, 1:500). Actin [(D18C11) cell signaling, cat #12748S] was used as a loading control.

### 2.10. Human Primary FTE Cells and p53-Inactivated FTE Cell Lines

Human primary FTE cells were processed and cultured according to Karst et al. [16]. FT246-p53 null FTE (shRNA knock-out) and Simian virus 40 large T-Antigen p53-inactivated human FTE cell lines FT190 and FT194 were obtained from Dr. Ronny Drapkin (Department of OBGYN, University of Pennsylvania, Biomedical Research, Philadelphia, PA, USA) [17].

### 2.11. Cell Culture and Western Blot Analysis

The primary FTE cells (FT1018, FT1019, FT1026 and FT1028) and the p53-inactivated FTE cell lines (FT190 and FT194) were cultured in DMEM/F12 supplemented with 0.5–2% Ultroser G (cat #67042 Crescent Chemical Company, Islandia, NY, USA) and 1% penicillin/streptomycin and treated with different concentrations of cholecalciferol (Chole), 25(OH)D3 or 1,25 (OH)2 D3(Calcitriol (CAL)) for 24–48 h on 6 cm cell-culture plates (Primaria Multi-well, Corning Co., Corning, NY, USA, #3295). At the end of each treatment, 20–40 μg of protein were used for standard western blotting analysis for cleaved caspase-3 (Asp175) (5A1E) Rabbit mAb, (Cell Signaling #9664-), CYP2R1 (Fisher Scientific, Hampton, NH, USA, #PIPA5101313), CYP27B1 (Fisher Scientific, #PIPA579128), CYP24A1 ((h87), Santa Cruz Biotechnology, Dallas, TX, USA, #sc-66851), vitamin D receptor (VDR-Cell Signaling, #12550S), and pan-actin ((D18C11) cell signaling, (cat #12748S). To quantify changes in protein expression, densitometry values of each protein were normalized to actin protein bands using LabWorks software (Version 4.6.00.0). FTE cell viability, cytotoxicity, and caspase activity were examined using ApoTox-Glo Triplex Assay (Promega, Madison, WI, USA, #G6321) according to the manufacturer’s instructions using 5000 cells per well on a 96-well white plate in phenol-free medium with different concentrations of vitamin D. Standard MTS assay was performed using 5000 cells/well for each cell type in phenol red-free media on a 96-well plate. The FT190 cell line was treated with CYP24A1 inhibitor-SDZ285428 (Product Code: TRC-S211113) (10 µM or 100 nM) alone or in combination with 10µM of Cholecalciferol for 24 h.

### 2.12. Scratch Assay

FT190 and FT194 cells were cultured in DMEM/F12 supplemented with 2% Ultroser G and 1% penicillin/streptomycin at 70% confluence in individual six well plates (Primaria Multi-well, Corning, #353846) and maintained in an incubator at 37 °C and 5% CO_2_ overnight. After the overnight incubation, a scratch/wound was generated with a sterile 200 µL pipette tip in the middle of each well that contained cells. A grid was drawn on the outside of the well to mark an area of the scratch for future measurements. The cell culture media was then aspirated, and the cells were treated with vehicle, 2.5 µM, 5 µM, or 10 µM of cholecalciferol (Sigma, #C1357) in DMEM/F12 supplemented with 0.5% Ultroser G and 1% penicillin/streptomycin. Using a Nikon Eclipse microscope outfitted with the NIS Element BR imaging program, images were acquired of the highlighted scratch, and 4–7 measurements of the scratch width were recorded at 0 h, 24 h, and 48 h post-treatment.

### 2.13. Statistical Analysis

Data comparisons were performed using two-tailed unpaired *t*-tests. *p* values of 0.05 or less were considered statistically significant. Statistical analysis was conducted using Windows, Microsoft Office Excel, 2019.

## 3. Results

**Figure 1:**
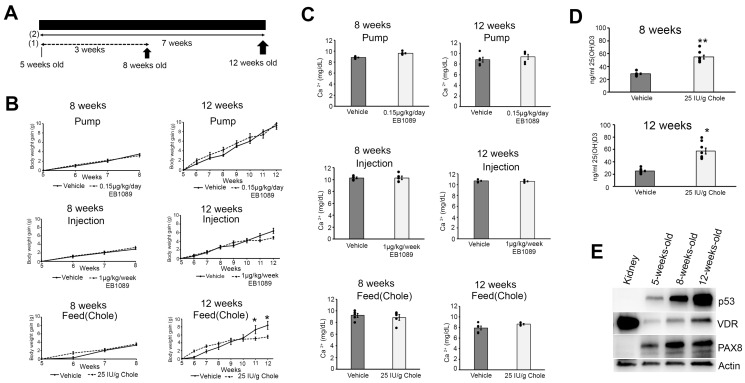
Effects of vitamin D on mogp-TAg mouse weight and calcium levels 8 and 12 weeks of age. (**A**) Trial design for the three different delivery systems for vitamin D in 5-week-old mice. Mice were euthanized at (1) 8 weeks of age (short term, 3 weeks of treatment) and (2) 12 weeks of age (long term, 7 weeks of treatment) for each delivery system. Sample size for each age group is *n* = 60 (a) Vehicle (*n* = 10) and osmotic mini-pump (0.15 µg/kg/day of EB1089) (*n* = 10), (b) Vehicle (*n* = 10) and I.P. injection (0.5 µg/kg EB1089 twice per week) (*n* = 10), and (c) Vehicle (*n* = 10) and feed (25 IU cholecalciferol (Chole) per gram of diet ad libitum) (*n* = 10). (**B**) Body weight gain (BWG) (grams) charts for the short- and long-term trials for each treatment group. (**C**) Calcium (Ca^2+^) (mg/dL) levels in the plasma of vehicle (*n* = 5) and vitamin D (*n* = 5) treated mice in the 3 different treatment groups at 8 and 12 weeks of age. (**D**) Plasma quantification of 25(OH)D3 at 8 weeks for vehicle (*n* = 6) and Chole (*n* = 8) and at 12 weeks for vehicle (*n* = 10) and Chole (*n* = 8). (**E**) Western blot of p53, VDR, and PAX8 in the kidney of 8-week-old mice and the fallopian tubes of 5-, 8- and 12-week-old mice. Mean ± SEM. * (*p* < 0.05), ** (*p* < 0.005), *t*-test.

To examine whether vitamin D inhibits fallopian tube (FT) carcinogenesis in the mogp-TAg mice, we performed short (3 weeks) and long-term (7 weeks) trials with vitamin D using three vitamin D delivery systems/formulations. For these experiments, 3 groups of 40 5-week-old female mogp-TAg mice were each divided into two cohorts of 20 mice. For each vitamin D delivery formulation, 10 vehicle-treated and 10 vitamin D-treated mice were euthanized (arrows) at 8 (short term [1]) and 12 weeks (long term [2]) of age (Figure 1A). As described in the methods section for vehicle (vitamin D solvent) and vitamin D delivery, the interventions were as follows: Trial 1 (ALZET osmotic pump-1,25(OH)2D3 analogue EB1089), Trial 2 (intraperitoneal (I.P.) injection-1,25(OH)2D3 analogue EB1089), and Trial 3 (cholecalciferol (Chole) in the feed).

### 3.1. Effects of Vitamin D on mogp-TAg Mouse Weight

As the mogp-TAg mice age, weight gain is a typical concomitant with an increase in the reproductive tract tumor burden and metastatic tumor growth on other organs [13,14]. Mouse weights were recorded weekly, starting at 5 weeks of age until the end of each trial. In the short-term vitamin D trial, there were no differences in weight gain between vehicle and vitamin D-treated mice, regardless of the delivery method (Figure 1B). However, in the long-term vitamin D trial, only the feed group had a significant reduction in body weight gain at 11 and 12 weeks (*p* < 0.05) compared to the vehicle group (Figure 1B).

### 3.2. Effects of Vitamin D Delivery on Calcium and 25(OH)D3 Levels

Since high levels of active vitamin D can cause hypercalcemia, which may have a negative impact on the mice, we measured plasma calcium levels at the end of each trial. There were no differences in the calcium levels at the 8- or 12-week time points in the pump, injection, or feed groups (Figure 1C). For the mice in the vitamin D feed group, 25(OH)D3 levels were measured at 8 and 12 weeks. As expected, supplementation of the diet with Chole significantly increased plasma 25(OH)D3 levels in the 8- (*p* < 0.005) and 12-week-old (*p* < 0.05) mice, respectively, (Figure 1D).

### 3.3. Expression of the Vitamin D Receptor (VDR) in the Fallopian Tube of mogp-TAg Mice

To examine whether the FT expresses VDR, western blot was performed on homogenates of whole mogp-TAg FTs at 5, 8, and 12 weeks of age. Homogenates of 8-week-old mogp-TAg mouse kidneys were used as a positive control for VDR. Based on western blot analysis, VDR is expressed in the FT in the mice at 5, 8, and 12 weeks of age (Figure 1E). PAX8 was used as a marker for the secretory cells in the epithelial layer of the FT. The kidney had undetectable levels of PAX8 protein (Figure 1E). Interestingly, as the mice aged from 5 to 12 weeks, there was a gradual increase in p53 protein expression (Figure 1E). This coincided with the evolution of p53 signatures to STIC and eventually to invasive carcinoma. The kidney had undetectable levels of p53 protein (Figure 1E).

### 3.4. Morphological Changes in the FT Following Treatment with Vitamin D

**Figure 2:**
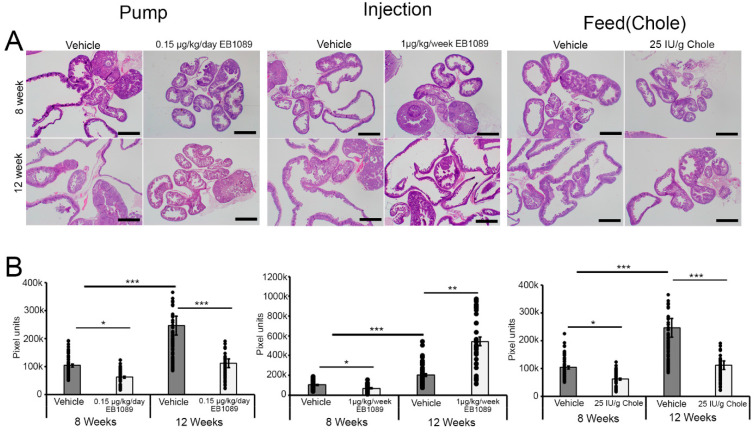
Morphological changes in the fallopian tube at 8 and 12 weeks of age following treatments. (**A**) Representative H&E sections of the FT in the vehicle- and vitamin D-treated mice at 8 and 12 weeks of age following treatments in the 3 groups. Images were captured at 2× magnification. Scale bar: 1 mm. (**B**) Average cross-sectional area (pixel units) of the FT in Pump group at 8 weeks (vehicle (*n* = 65) and EB1089 (*n* = 47) and 12 weeks (vehicle (*n* = 57) and EB1089 (*n* = 45), Injection group at 8 weeks (vehicle (*n* = 52) and EB1089 (*n* = 65) and 12 weeks (vehicle (*n* = 55) and EB1089 (*n* = 39) and feed group at 8 weeks (vehicle (*n* = 60) and Chole (*n* = 61) and 12 weeks (vehicle (*n* = 55) and Chole (*n* = 54). mean ± SEM, * (*p* < 0.05), ** (*p* < 0.005), *** (*p* < 0.0005), *t*-test.

As mogp-TAg mice age, there is a significant increase in the volume/cross-sectional area of the fallopian tube [14]. In our previous report [15], we showed that treatment with the progestin-depot medroxyprogesterone acetate (DMPA) significantly reduced the cross-sectional area of the fallopian tube in the mogp-TAg mice by inhibiting the expansion of p53 signatures, STIC lesions, and cancer in the FT epithelial layer. To examine whether vitamin D had a similar effect on the FT, we measured the cross-sectional area (pixel units) of the vehicle and the vitamin D treatment groups at 8 and 12 weeks. The FT cross-sectional area was significantly reduced in the mice treated with vitamin D at 8 weeks via pump, injection, and feed compared to vehicle (*p* < 0.05) (Figure 2A,B). Similarly, the FT cross-sectional area was significantly smaller in the pump and feed groups at 12 weeks when compared to the vehicle (*p* < 0.0005). Interestingly, the cross-sectional area in the vitamin D injection group was significantly larger at 12 weeks compared to the vehicle group (*p* < 0.05) (Figure 2A,B). In these mice, the FTs were grossly enlarged and filled with fluid; this was not observed in the vehicle group (Figure S1). Since the enlarged fluid-filled FT was only observed in the injection group at 12 weeks, we speculated that this is an adverse event that could have resulted from the bolus administration of vitamin D to the mice. Further experiments will be needed to understand this phenomenon in the fallopian tube. However, that is beyond the scope of this study.

### 3.5. Vitamin D Inhibits p53 Signatures and STIC Incidence in the FT

**Figure 3:**
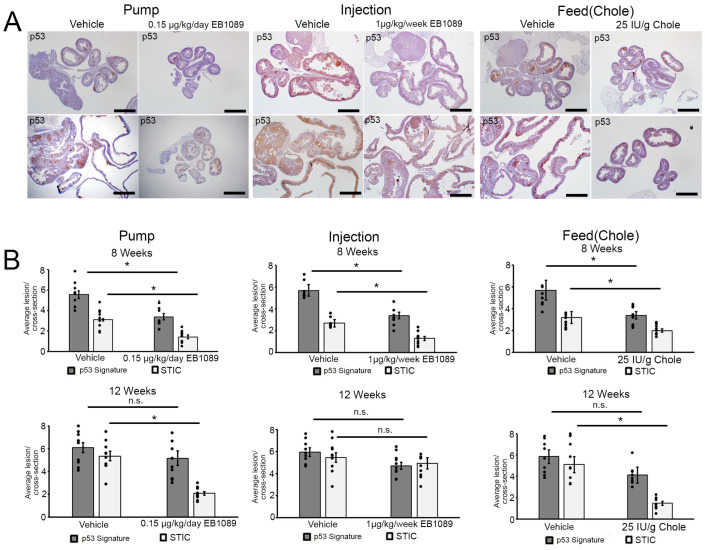
p53 positivity in the FT at 8 and 12 weeks of age following treatments. (**A**) Representative IHC staining of p53 at 8 and 12 weeks following treatment with vehicle and vitamin D in the 3 treatment groups. Images were captured at 2× magnification. Scale bar: 1mm. (**B**) Quantification of the p53 signatures and STIC lesions in the FT following treatment with vehicle (*n* = 10) and vitamin D (*n* = 10) at 8 at 12 weeks in the pump, injection, and feed treatment groups. Not significant (n.s.), mean ± SEM, * (*p* < 0.05), *t*-test.

FT sections stained immunohistochemically for p53 were used to quantify the number of p53 signatures and STICs following treatments at 8 and 12 weeks (Figure 3A). On average, as compared to the vehicle group, there were significantly fewer p53 signatures and STICs in the vitamin D-treated mice at 8 weeks, regardless of the vitamin D formulation or method of delivery. For the average number of lesions per FT cross-section per treatment group, there were six p53 signatures and three STIC lesions in the vehicle group at 8 weeks for the pump, injection, and feed (*n* = 10 for each group). In contrast, there were three p53 signatures and one STIC lesion in the vitamin D pump group (Figure 3B, *p* < 0.05), three p53 signatures and one STIC lesion in the vitamin D injection group (Figure 3B, *p* < 0.05), and three p53 signatures and two STIC lesions in the vitamin D feed group (Figure 3B, *p* < 0.05). In the long-term trial (12-week-old mice), as compared to the vehicle, regardless of the vitamin D-delivery system, there was no significant difference in the number of p53 signatures (Figure 3B). However, there were significantly fewer STICS in the groups receiving vitamin D via pump (EB1089) or feed (Chole) (Figure 3B, *p* < 0.05). No significant difference was noted in the mice receiving vitamin D via injection (Figure 3B). Interestingly, there were 50% fewer STIC lesions in the vitamin D feed group compared to the vitamin D pump group at 12 weeks, suggesting that although both vitamin D formulations inhibit p53 signatures from maturing into STIC, cholecalciferol delivered in the food may be more efficacious than continuous administration of an active form of vitamin D.

### 3.6. Vitamin D Inhibits Lesion Formation and Proliferation in the FT

**Figure 4:**
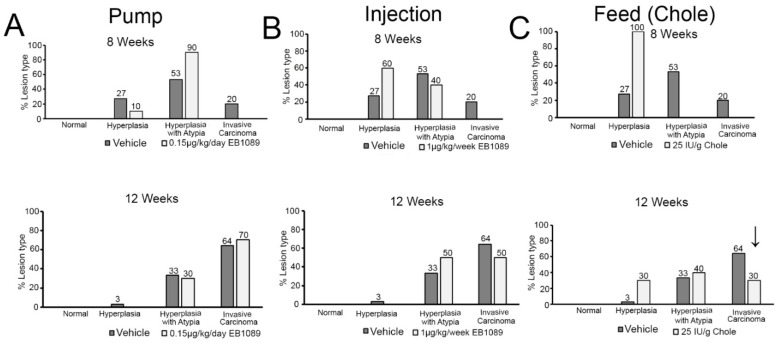
FT histologic/pathologic characterization for normal, hyperplasia, hyperplasia with atypia, and invasive carcinoma at 8 and 12 weeks of age following treatments. The percentage of mice with the different types of FT lesions at 8 and 12 weeks are displayed on the graph: (**A**) The pump treatment group. (**B**) The injection treatment group. (**C**) The feed treatment group. Arrow indicates a 50% reduction in invasive carcinoma between vehicle and Chole in the feed group at 12 weeks.

Fallopian H&E sections were examined in a blinded fashion by two veterinary pathologists and characterized as normal, or containing hyperplasia, hyperplasia with atypia, or invasive carcinoma. In the short-term 3-week trial (8-week-old mice), 27% of the vehicle-treated mice developed hyperplasia, 53% developed hyperplasia with atypia, and 20% developed invasive carcinoma in the FTE (Figure 4A–C). In the pump group (EB1089), 10% developed hyperplasia and 90% of the mice developed hyperplasia with atypia in the FTE. In the injection group (EB1089), 60% had hyperplasia and 40% of the mice developed hyperplasia with atypia in the FTE. All of the mice in the feed group (Chole) developed hyperplasia; none had atypia or cancer (Figure 4A–C). In the long-term 7-week trial (12-week-old mice), 3% of the vehicle-treated mice developed hyperplasia, 33% developed hyperplasia with atypia, and 64% developed invasive carcinoma in the FTE (Figure 4A–C). In the pump group, 30% developed hyperplasia with atypia and 70% of the mice developed invasive carcinoma in the FTE. In the injection group, 50% had hyperplasia with atypia and 50% of the mice developed invasive carcinoma in the FTE. In contrast, in the feed group at 12 weeks, 30% developed hyperplasia, 40% developed hyperplasia with atypia, and 30% of the mice developed invasive carcinoma (Figure 4A–C). Ultimately, in the short-term trials, vitamin D inhibited carcinogenesis in the fallopian tube regardless of the mode of delivery or formulation by shifting the FTE histology toward a more benign phenotype. In the long-term trials, only vitamin D delivered as cholecalciferol in the food inhibited invasive carcinoma, conferring a 50% reduction in fallopian tube cancer when compared to the vehicle group (Figure 4C). This observation suggests that vitamin D administered in the food as cholecalciferol was the most effective modality for inhibiting FT cancer.

It has been previously shown that p53-positive cells in the FT co-localize to areas with increased cellular proliferation [14,15]. Since we observed that vitamin D administered in the food significantly reduced the number of p53-positive cells in the FTE by clearing p53 signatures, STICs, and cancer, we sought to examine whether cholecalciferol might inhibit FT cell proliferation. As shown in Figure S2A,B, as the p53-positive cells were cleared, we observed a reduction in Ki67-positive cells (arrowhead) in the epithelial layer at 8 and 12 weeks in the feed group compared to vehicle-treated mice.

### 3.7. Vitamin D Lowers CA125 Levels in the mogp-TAg Mice

The ovarian cancer tumor marker CA125 is known to strongly correlate with disease burden in most women with ovarian cancer. Additionally, it has been shown to decrease in the 7, 12 dimethylbenz [a] anthracene-inducible ovarian cancer mouse model after treatment with vitamin D [18]. Here, we examine whether vitamin D had an effect on CA125 levels in all the treatment groups at 8 and 12 weeks. As shown in Figure S3A–C, there was a significant increase (*p* < 0.005) in the level of CA125 from 8 to 12 weeks in all of the vehicle-treated mice. As compared to vehicle-treated mice, there were no differences in CA125 levels in mice with EB1089 delivered via pump at 8 and 12 weeks (Figure S3A). The EB1089-injection group had significantly lower CA125 levels at 8 weeks (*p* < 0.05) when compared to the vehicle, while there were no differences at 12 weeks (Figure S3B). In contrast, the feed (Chole) group had significantly lower CA125 levels as compared to vehicles at both 8 (*p* < 0.05) and 12 (*p* < 0.005) weeks (Figure S3C). Notably, the decrease in CA125 levels was consistent with the decline in lesion formation and cancer incidence. Since it appeared that cholecalciferol administered in the food might be the most effective modality for shifting the FT in mice to a more benign phenotype, we further characterized this treatment group to better understand the mechanisms involved in inhibiting FT carcinogenesis.

### 3.8. Cholecalciferol Induces Apoptosis in the FT

**Figure 5:**
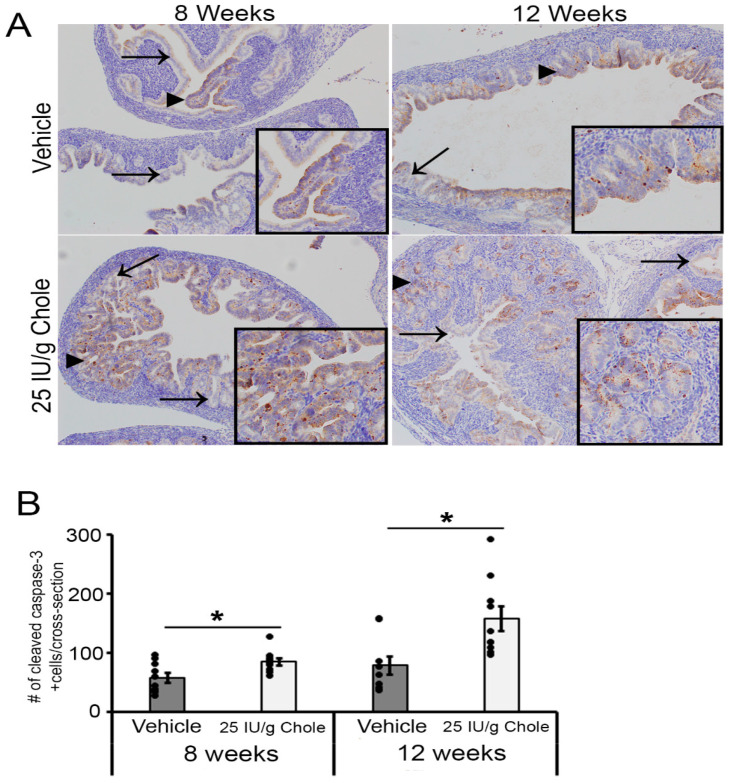
Cleaved caspase-3 induction in the FT at 8 and 12 weeks of age after treatment with Cholecalciferol. (**A**) Representative FT IHC of cleaved caspase-3 (brown) captured at 10× magnification following treatment with vehicle and Chole at 8 and 12 weeks. Triangles highlight cropped FTE layer (black squares) showing cleaved caspase-3 positive cells. Arrow indicates normal-appearing epithelial layer that does not contain cleaved-caspase 3 positive cells. (**B**) Quantification of cleaved caspase-3 positive cells in the FT cross-section following treatment at 8 weeks for vehicle (*n* = 71) and Chole (*n* = 55) and at 12 weeks for vehicle (*n* = 52) and Chole (*n* = 65). mean ± SEM, * (*p* < 0.05), *t*-test. *n* = the number of cross-section examined for cleaved caspase-3 positive cells.

It is well-documented that vitamin D can induce apoptosis in cancer cells [19,20,21]. To investigate whether cholecalciferol can induce apoptosis in the FTE of the mogp-TAg mice, IHC for cleaved caspase-3 was performed on adjacent sections that were used for p53 staining to examine FTE cell death in cross-sections from the feed group cohort at 8 and 12 weeks. At both 8 and 12 weeks, compared to the vehicle, there were significantly more cleaved caspase-3 immuno-reactive cells in the cholecalciferol group (Figure 5A,B) (*p* < 0.05). Similar to what we had shown in our previous study with progestin in mogp-TAg mice [15], there was cholecalciferol-induced cell death in FTE cell clusters that morphologically resembled p53 signatures and STIC lesions (triangles). Cleaved caspase-3 immunoreactivity was not observed in FTE cells that appeared normal (Figure 5A) (arrows). These data suggest that cholecalciferol induces apoptosis in abnormal FTE cells, and that this might be a mechanism that is involved in clearing genetically compromised cells from the FTE.

### 3.9. Cholecalciferol Induces Apoptosis and Reduces Cell Viability and Proliferation in Fallopian Tube Cell Cultures

Having seen robust inhibition of carcinogenesis by cholecalciferol in vivo, we sought to further characterize the mechanism(s) underlying this biologic effect. Cholecalciferol does not directly activate vitamin D-response genes. Rather, it must be converted first to the active form of vitamin D [1, 25(OH)2 D3 (calcitriol)], which then elicits a biological effect by binding to the vitamin D receptor (VDR), which then activates vitamin D-related downstream events. Because renal production of calcitriol is highly controlled, with minimal variation even in the setting of high intake of cholecalciferol, we hypothesized that a robust chemopreventive effect of cholecalciferol in vivo in fallopian tube cells would require that it both undergo conversion to calcitriol in the FTE and that the FTE be responsive to calcitriol. First, we investigated whether primary and p53-inactivated FTE cells are responsive to calcitriol. Treatment of primary FT cells (FT1018 and FT1019) and p53-inactivated cell line FT190 with calcitriol in culture led to the induction of vitamin D-responsive genes Calmin (CLMN) [22], VDR, and cytochrome P450 family 24 subfamily A member 1-CYP241 (enzyme used to inactivate calcitriol) (Figure S4Ai and S4Aii, respectively). Next, we observed that treatment of FT190 with either cholecalciferol or 25(OH)D3 induced the same calcitriol-responsive genes (Figure S4Aiii and S4Aiv, respectively). Both Chole and 25(OH)D3 induced the expression of CYP27B1 in p53-inactivated cell lines FT190 and FT194 (Figure S4B). It has been shown that vitamin D can affect the expression of p53 in cancer cells [23]. Therefore, we examined whether vitamin D affected the expression of p53 in FT190 and FT194. Even though Chole, 25(OH)D3 and CAL caused an induction of VDR in FT190 and FT194, we did not observe any robust changes in p53 protein expression (Figure S4Ci,ii). These observations suggest that FTE cells have the cellular mechanism to synthesize calcitriol from cholecalciferol and that vitamin D does not affect p53 expression in FT190 and FT194. Interestingly, when we examined the expression of CYP2R1 and CYP27A1 in the liver and CYP27B1 and VDR in the kidney of 8- and 12-week feed trial mice, we did not observe any significant protein increases in the feed group compared to the vehicle (Figure S5A–D). Expression of these enzymes is tightly regulated in the liver and kidney, explaining the lack of any significant change in expression in these proteins despite a significant increase in plasma 25(OH)D3.

**Figure 6:**
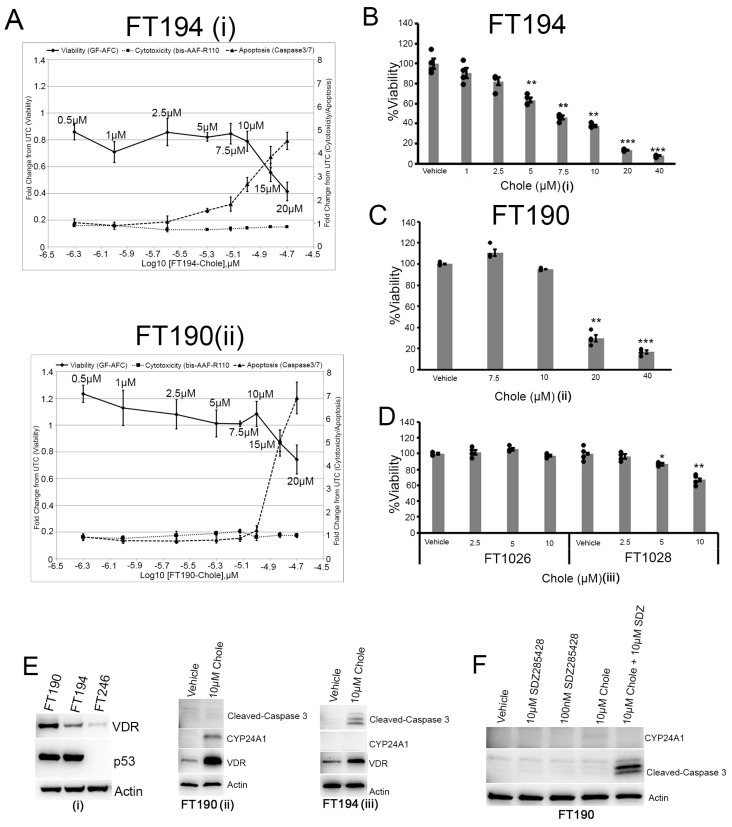
Cholecalciferol induces apoptosis and inhibits proliferation of FTE cells expressing inactivated p53 protein. (**A**) Apotox-Glo Triplex Assay showing cell viability (solid line), cytotoxicity (broken line), and apoptosis (dotted line) at 0.5 µM, 1 µM, 2.5 µM, 5 µM, 7.5 µM, 10 µM, and 20 µM Chole in FT194 (i) and FT190 (ii). Each concentration was done in triplicates. (**B**) MTS assay showing dose-dependent growth inhibition of FT194 at 24 h of treatment with increasing dose of Chole; the average of four independent wells was used to obtain the absorption for each concentration. Mean ± SEM, ** (*p* < 0.005), *** (*p* < 0.0005), *t*-test. (**C**) MTS assay showing growth inhibition of FT190 at 24 h of treatment with increasing dose of Chole; the average of four independent wells was used to obtain the absorption for each concentration. Mean ± SEM, ** (*p* < 0.005), *** (*p* < 0.0005), *t*-test. (**D**) MTS assay in primary FT cells (FT1026 and FT1028) showing the percentage growth inhibition with increasing dose of Chole; the average of four independent wells was used to obtain the absorption for each concentration. Mean ± SEM, * (*p* < 0.05), ** (*p* < 0.05), *** (*p* < 0.005), *t*-test. (**E**) Western blot of endogenous p53 in FT190, FT194, and FT246 (negative) (i), in addition to cleaved-caspase 3, CYP24A1, and VDR following treatment with vehicle and Chole in FT190 (ii) and FT194 (iii). (**F**) Western blot of cleaved-caspase 3 and CYP24A1 following treatments CYP24A1 inhibitor SDZ285428 only, Chole only, and SDZ285428/Chole combination in FT190.

Next, we tested the effect of cholecalciferol on SV40-transformed fallopian tube cells (FT194 and FT190) to see if there was a similar effect to what we observed in the mogp-TAg mice. Chole has been shown to reduce cell viability and induce apoptosis in CaSki cervical tumor cells [24]. Therefore, we examined whether Chole could exert similar effects in FT190 and FT194. As shown in Figure 6A,B, using the Apotox-Glo Triplex assay, cholecalciferol induced apoptosis and inhibited cell viability in FT194 and FT190. However, it required higher concentrations of Chole to induce apoptosis in FT190. According to MTS assay data, Chole inhibited FT194 proliferation in a dose-dependent manner (Figure 6B) with an IC_50_ value of 8.5µM. Since it required higher concentrations of Chole to induce apoptosis or inhibit cell proliferation using Apotox-Glo, we performed an MTS assay in FT190 starting at 7.5µM of Chole. While 10µM of Chole had a significant effect on inhibiting FT194 proliferation, this dose had no effect on FT190 proliferation. Based on the growth inhibition of FT190 by 20µM Chole, we estimated the IC_50_ of about 18µM for Chole in FT190 (Figure 6C). These results were further validated with the scratch-/wound-healing assay showing that chole can inhibit the proliferative capacity of FT194 and FT190. However, there was a more robust effect in retarding the closing of the scratch in FT194 compared to FT190 (Figure S6A–D). Interestingly, primary FTE cells were significantly less sensitive to Chole inhibition of cell proliferation (Figure 6D) compared to FTE cells with inactivated p53. Before conducting the next set of experiments, we performed western blots for p53 expression in FT190, FT194, and FT246 (p53 null). As shown in Figure 6Ei, FT190 and FT194 expressed p53 while FT246 was null. All three cell lines expressed the endogenous vitamin D receptor. From the Apotox-Glo and scratch assays, it appeared that FT194 was more sensitive to cholecalciferol than FT190. Treatment of FT190 with 10µM Chole caused an induction of CYP24A1 protein expression with almost no induction of cleaved-caspase 3, while in FT194, there was a significant induction of cleaved-caspase 3 and no induction of CYP24A1 (Figure 6Eii and Figure 6Eiii, respectively). Vitamin D receptor expression was induced in both cell lines. This suggested that the induction of CYP24A1 in FT190 might be inactivating the calcitriol synthesized from Chole and limiting its biological function. To test this hypothesis, we inhibited CYP24A1 in FT190 with SDZ285428 and then treated these cells with 10µM of cholecalciferol to see if resistance to apoptosis could be reversed. As shown in Figure 6E, the inhibition of CYP24A1 following treatment with cholecalciferol resulted in the induction of cleaved-caspase 3. Taken together, our in vivo and in vitro data suggest that cholecalciferol confers a chemopreventive effect by preferentially targeting abnormal FTE cells via apoptosis, reduction of cell viability, and reduction of proliferative capacity while sparing normal FTE cells. Alterations that may be more typical late in the course of carcinogenesis in FTE or ovarian cancer cells such as activation/overexpression of CYP24A1 can potentially abrogate the cancer-preventive effects of vitamin D. It is intriguing that the inhibition of CYP24A1 may sensitize the FTE to cholecalciferol-induced cell death.

## 4. Discussion

To our knowledge, this is the first study in vivo demonstrating a direct chemopreventive effect of vitamin D on fallopian tube carcinogenesis. As previously shown by our group and others, secretory cells in the fallopian tubes of mogp-TAg transgenic mice accumulate cells with SV40 inactivated p53 as early as 5 weeks of age. The p53-positive cells are focal and scattered in 5-week-old mice but evolve into p53 signatures and STICs by 7–8 weeks of age, with progressive neoplastic transformation and invasive cancers as the mice age. The prevalence of these lesions decreased significantly with vitamin D treatment, both via parenteral administration of active vitamin D analogue EB1089, as well as via administration of cholecalciferol in the diet, although the effect of cholecalciferol appeared more robust. Notably, similar to what we had shown previously with the progestin depo-medroxyprogesterone acetate (DMPA) [15], vitamin D treatment not only prevented neoplasia but significantly eradicated the early, histologically normal-appearing secretory cells harboring inactivated p53, targeting the earliest putative transformative event in the fallopian tube and thus ovarian carcinogenesis. Additionally, it is remarkable that in cells derived from the human FTE, we observed in vitro that vitamin D not only inhibited cell viability and induced apoptosis but also that the human FTE cells have the capability to synthesize the active form of vitamin D from cholecalciferol and 25 (OH)D3. These data strongly support the hypothesis that vitamin D has merit for the chemoprevention of ovarian cancer.

Due to the very broad range of biological effects attributable to vitamin D that can impact molecular pathways related to cancer, as well as the ubiquitous expression of the vitamin D receptor in most epithelia, it is plausible that a vitamin D-based strategy can be formulated for the effective chemoprevention of cancer. Notably, numerous reported antineoplastic effects of vitamin D have been shown in vitro and in vivo, including the inhibition of proliferation, enhancement of apoptosis, induction of differentiation, inhibition of cancer stem cells and epithelial–mesenchymal transition, and inhibition of inflammation, antioxidant effects, and immune modulation. In addition, vitamin D has been shown to inhibit angiogenesis and cancer cell invasion and metastasis [9,25,26,27]. These effects of vitamin D would be expected to arrest or reverse not only the early but also the latter stages of carcinogenesis, with the potential for both the prevention and treatment of cancer.

Yet, surprisingly, population-based studies investigating the relationship between vitamin D status and cancer outcomes have yielded inconsistent results, with some demonstrating a vitamin D benefit and others reporting null associations [25]. These conflicting results have applied broadly to most cancers, including ovarian cancer [28], although the largest body of evidence has applied to the more commonly occurring cancers such as colon, breast, and prostate. More recently, randomized clinical trials have similarly failed to convincingly demonstrate that vitamin D supplementation impacts cancer incidence or mortality. These inconsistent and/or null results have led some to conclude that vitamin D has little utility as a chemopreventive or treatment intervention for cancer [29].

However, the conflicting results to date could be attributable to a number of methodological limitations and/or confounding factors that have not been completely accounted for and which are particularly relevant to the study of vitamin D. For example, ecological studies using geographic location as a proxy for sunlight and, thus, vitamin D exposure lack the incorporation of important individual data such as the use of supplements and the amount of outdoor activity, as well as the extent of prior residence in other geographic locations. For observational studies using plasma 25(OH)D3 levels as a proxy for the status of vitamin D nutrition, variance in the quality of 25(OH)D3 measurements, or fluctuations in 25(OH)D3 levels impacted by seasonality, could have influenced the results. Additionally, 25(OH)D3 measurements taken near the time of diagnosis may be impacted by factors such as underlying disease status. More recently, Mendelian randomization has gained popularity as potentially being less biased as the approach reduces both reverse causation and confounding that can occur in epidemiologic studies. In this approach, a genetic variation or trait, usually a single nucleotide polymorphism (SNP) that is associated with the exposure of interest and independent of confounding factors, is used as a proxy for the exposure to examine the effect of the exposure on an outcome. For example, SNPs strongly associated with vitamin D metabolism (and thus higher or lower 25(OH)D3 levels) can be used to assess the causality of vitamin D exposure to cancer incidence or mortality. Mendelian studies are also subject to their own limitations, though, as significant associations can be missed if the sample size is too small or only a very limited number of SNPs are used that explain a small proportion of the variation in the risk factor (in this case, 25(OH)D3). In addition, faulty conclusions can occur if the genetic variant chosen influences the outcome measure independent of the hypothesized exposure (pleiotropy). Interestingly, in the largest study published to date, including 74 SNPs that collectively could explain only 4% of the phenotypic variation of 25(OH)D3 levels, the results were null across a broad range of cancers but significant for ovarian cancers, suggesting an 11% reduction in ovarian cancer risk for every one standard deviation rise of 25(OH)D3 [30].

Several recently published large randomized clinical trials (RCTs) concluded that there was no impact of vitamin D supplementation on cancer outcomes. However, study-design limitations may have impacted the ability to show a beneficial impact of vitamin D [26]. In all, baseline 25(OH)D3 levels in the study cohorts were consistent generally with vitamin D sufficiency, with only a small proportion of subjects being vitamin D deficient. Thus, the study populations may have been less likely to fully benefit from vitamin D supplementation. The studies were not specifically designed for vitamin D supplementation to target a specific 25(OH)D3 level, and study lengths may not have been sufficient [31]. However, on close inspection of the clinical trial data, some important insights that are supportive of vitamin D can be gleaned. In the VITAL study, which is the largest RCT trial to date, and which concluded no beneficial effect of cholecalciferol on cancer incidence or mortality, exclusion of the first 2 of 5 years of follow-up revealed a statistically significant 21% reduction in cancer-related mortality. Further, in subjects of normal weight (BMI of 25 or less), vitamin D supplementation conferred a statistically significant 24% reduction in cancer incidence and a 42% reduction in cancer mortality [31,32]. Two-thirds of subjects in the VITAL study were overweight (40.5%) or obese (27%). Notably, in the overweight or obese population, baseline 25(OH)D3 was lower, and although 25(OH)D3 levels increased significantly for all subjects receiving cholecalciferol in the study, the increase was blunted in the subjects who were overweight. Thus, overweight individuals likely require more vitamin D to achieve its beneficial effects. In the study by Lappe et al., supplementation with Chole and calcium failed to impact cancer outcomes [33]. Yet, similar to the VITAL study, after the exclusion of subjects who developed cancer in the 1st year and who, thus, may have had occult cancers at the start of the trial, those taking vitamin D had a 35% reduction in cancer incidence. Additionally, a similar benefit to vitamin D was noted when comparing baseline levels of 25(OH)D3 below 30 ng/mL to those between 30 and 55 ng/mL. The proper design of vitamin D supplement trials will likely need to adhere to the criteria proposed for RCTs of nutrients, including careful measurements and the use of baseline 25(OH)D3 for enrollment, dosing of vitamin D to sufficiently raise 25(OH)D3 levels, and careful control of use of vitamin D supplements [34]. It would also be ideal to dose vitamin D to a target 25(OH)D3 level, as there is significant variability in individual vitamin D responsiveness [35].

Further studies will be required to determine the optimal vitamin D formulation, dosing, and schedule for effective chemoprevention of ovarian cancer. Although our results suggest a more robust effect of cholecalciferol than an analogue of the active form of vitamin D (EB1089), we cannot exclude the possibility that EB1089 appeared less effective either due to rapid metabolism or a less-than-optimal dose used. Notably, in our study, we did not see a beneficial effect of non-daily, bolus administration of vitamin D. This finding is similar to that of the recently published ViDA [36] and D-Health [37] randomized trials, although in those trials, in contrast to our study, the intermittent, monthly formulation of dosing of vitamin D that was administered was cholecalciferol and not an active form of vitamin D. Notably, we found the most effective intervention was cholecalciferol administered daily in the diet. This is consistent with the subgroup results of the VITAL and Lappe trials described above, where cholecalciferol was administered daily, and with a meta-analysis of RCTs in 2019 suggesting a reduction in cancer mortality in those subjects who had a longer-term follow-up [38].

In contrast to calcitriol or analogues of calcitriol, which can cause hypercalcemia, cholecalciferol is safe, non-toxic, and inexpensive, which are characteristics that would be ideal for chemoprevention. Notably, the renal 1 alpha-hydroxylase (1α-OHase [CYP27B1]) enzyme is tightly regulated physiologically via feedback loops related to circulating levels of calcium, PTH, and 1,25(OH)2D3 to limit the risk of hypercalcemia. In contrast, the 1α-OHase that is expressed in many non-renal tissues, including cells from the immune system and many epithelia, including epithelia from the gynecologic tract, is not tightly regulated and operates below its Michaelis constant. Higher concentrations of circulating 25(OH)D3 could, therefore, drive increased peripheral, non-renal production of 1,25(OH)2D3 with local levels that could be higher than systemic [10,39,40,41]. It is thus likely that the inhibitory effect on FTE carcinogenesis that we observed with cholecalciferol in vivo was due to the localized production of 1,25(OH)2D3 in the FTE, with subsequent autocrine or paracrine chemopreventive effects in FTE cells by locally produced 1,25(OH)2D3. This is further supported by our in vitro results demonstrating that FTE cells express both CYP2R1 and CYP27B1 and undergo activation of vitamin D-responsive genes in response to both cholecalciferol and 25(OH)D3. In our study, the mice on cholecalciferol had steady-state 25(OH)D3 levels of just under 60 ng/mL. It is interesting to speculate whether higher doses of cholecalciferol and, thus, higher plasma 25(OH)D3 levels could confer greater chemopreventive effects against FTE carcinogenesis, in that the optimal dosing for chemoprevention may be higher than that for other physiologic effects of vitamin D, such as the promotion of bone health.

The biological mechanisms underlying the vitamin D-mediated clearance of dysplastic cells in the fallopian tube remain to be fully elucidated. Our findings suggest that vitamin D activates apoptosis. In the mogp-TAg mouse, we observed vitamin D-induced apoptosis in areas of the FTE that contained p53 signatures, STICs, and carcinoma, all of which co-localized with cellular alteration in p53. This was confirmed in vitro in p53-inactivated fallopian tube cell cultures. Mutant p53 has been shown to interact with the VDR and associate with a number of vitamin D-response elements [42]. Whether or not the induction of apoptosis in the FTE by vitamin D is at least in part a p53-mediated event remains to be determined. Paradoxically, mutant p53 has been shown to reverse the pro-apoptotic effect of vitamin D in a number of cell lines, including those derived from the colon, breast, and prostate [42]. In contrast, in ovarian cancer, where p53 is the most common genetic alteration, a number of in vitro studies have shown an apoptotic effect of vitamin D. Notably, in ovarian cancer cell lines, vitamin D has been shown to have an apoptotic effect that is p53-independent via the activation of caspase 9 and the downregulation of telomerase, either via decreasing its stability or via suppressed expression induced by miR-498 [9]. Additionally, vitamin D has also been shown to inhibit the growth of ovarian cancer cells via cell cycle arrest through VDR-mediated p53-independent induction of GADD45 [43,44], and, in our study, we found that vitamin D inhibited the proliferation of FTE cells. These non-p53-dependent mechanisms may further underlie vitamin D-related inhibition or clearance of dysplastic FTE cells and possibly overcome potential vitamin D resistance related to mutant p53. Alternatively, resistance to vitamin D-mediated apoptosis in FTE cells harboring mutant p53 may require the addition of other secondary mutations that are acquired later in the course of carcinogenesis and not yet present in early transformative events in the FTE.

The strengths of our study include the use of a valid transgenic animal model that recapitulates the early molecular events and genomics of fallopian tube and ovarian cancer. Notably, in the mogp-TAg mouse model, the alteration in p53 is an early event, similar to that in human fallopian tube carcinogenesis. The p53 alterations occur in PAX8-positive secretory FTE cells with a subsequent evolution of p53 signatures, STICs, and cancers similar to that in human fallopian tubes and ovarian cancers. The finding that vitamin D specifically cleared FTE cells with altered p53 via apoptosis, similar to what we previously showed in the same animal model with the ovarian cancer preventative progestin, further validates the results. Additionally, our in vivo results were further corroborated by in vitro results not only demonstrating a similar biological effect of vitamin D but also providing evidence for the novel finding that FTE cells have the capability to produce the active form of vitamin D from circulating precursors, specifically cholecalciferol, the form of vitamin D administered in the mouse diet. Therefore, these in vitro results provided mechanistic support for the chemopreventive effect in the mice administered the vitamin D in the diet. Further, the examination of mouse histology and pathology occurred in a blinded fashion, including the characterization of lesions by veterinary pathologists. Limitations of our study include that the transformation of the FTE in the mogp-TAg mouse is driven by SV40. Although SV40 confers alteration in p53, it may also induce other molecular alterations that may not be relevant to human ovarian carcinogenesis. Additionally, the dose chosen for the vitamin D analogue EB1089 may not have been optimal, and pharmacokinetics of EB1089 may not have been completely reflective of what we may have shown with calcitriol.

Moving forward, studies should seek to identify the optimal plasma 25(OH)D3 level to achieve a maximal chemopreventive effect against fallopian and ovarian carcinogenesis. Further, it will be important to test whether the optimal 25(OH)D3 target is higher in the setting of obesity. These data would ultimately inform the choice of cholecalciferol dosing for a prospective chemoprevention trial designed with adherence to ideal criteria for randomized critical trials of nutrients in women at increased risk of ovarian cancer. Finally, although our results suggest that the activation of apoptosis is an important mechanism underlying the chemopreventive effect of vitamin D on fallopian tube carcinogenesis, further studies should be performed to assess broader genomic, proteomic, or metabolomic effects of vitamin D in fallopian cells in vitro and in vivo to search for other important targets of vitamin D that can be optimized for chemoprevention.

## 5. Conclusions

Our study is the first to show, in a valid animal model for ovarian cancer, that vitamin D inhibited fallopian tube/ovarian carcinogenesis. Our results are strengthened by the finding that vitamin D eradicated fallopian tube epithelial cells harboring alterations in p53, the earliest putative genomic alteration in fallopian tube carcinogenesis. Among the different formulations we tested, cholecalciferol appeared to confer the most robust benefit. Our results suggest that circulating cholecalciferol and vitamin D prohormone 25(OH)D3 are converted by FTE cells to the active form of vitamin D, where it likely has a local autocrine or paracrine effect to mitigate carcinogenesis. Taken together, our data suggest that vitamin D has merit for further investigation as a chemopreventive for ovarian cancer.

## Data Availability

The data generated in this study are available upon request from the corresponding author.

## Supplementary Materials

The following supporting information can be downloaded at: https://www.mdpi.com/article/10.3390/nu16193318/s1, Figure S1. Effects of IP Injection of 1,25(OH)2D3 analogue EB1089 on mogp-TAg mouse FT at 12 weeks of age. Figure S2. p53 and Ki67 positivity in the FT at 8 and 12 weeks of age in the feed group. Figure S3. CA125 measurements in vehicle and vitamin D group at 8 and 12 weeks of age. Figure S4. Cholecalciferol activates vitamin D-responsive genes in vitro, but does not alter p53 expression. Figure S5. Protein expression of cytochrome P450 enzymes involved in the conversion of cholecalciferol to active vitamin D in the liver and kidney in the feed trial. Figure S6. Effect of cholecalciferol on scratch/wound healing assay.
