# Supplementary material for: Vitamin D Significantly Inhibits Carcinogenesis in the Mogp-TAg Mouse Model of Fallopian Tube Ovarian Cancer
Source: Nutrients. 2024 Sep 30;16(19):3318. doi: 10.3390/nu16193318 (PMC11478811; doi:10.3390/nu16193318)
Supplement: Supplementary file 1 [file nutrients-16-03318-s001.zip › nutrients-3203776-supplementary.pdf]

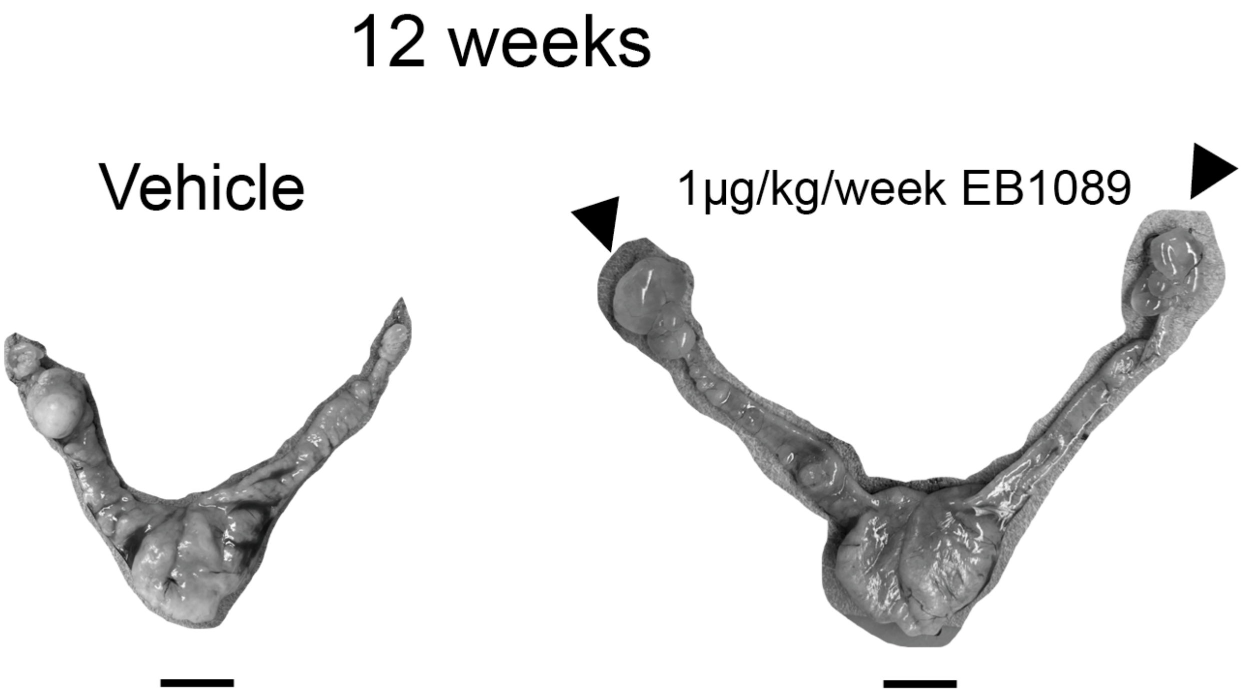

Figure S1. Effects of IP Injection of 1,25(OH)2D3 analogue EB1089 on mogp-TAg mouse FT at 12-weeks of age. Representative gross reproductive tract of the mogp-TAg mouse following injection of vehicle and EB1089 at 12 weeks of age. Triangles highlight fluid filled FT in the EB1089 group. Scale bar: 5mm.

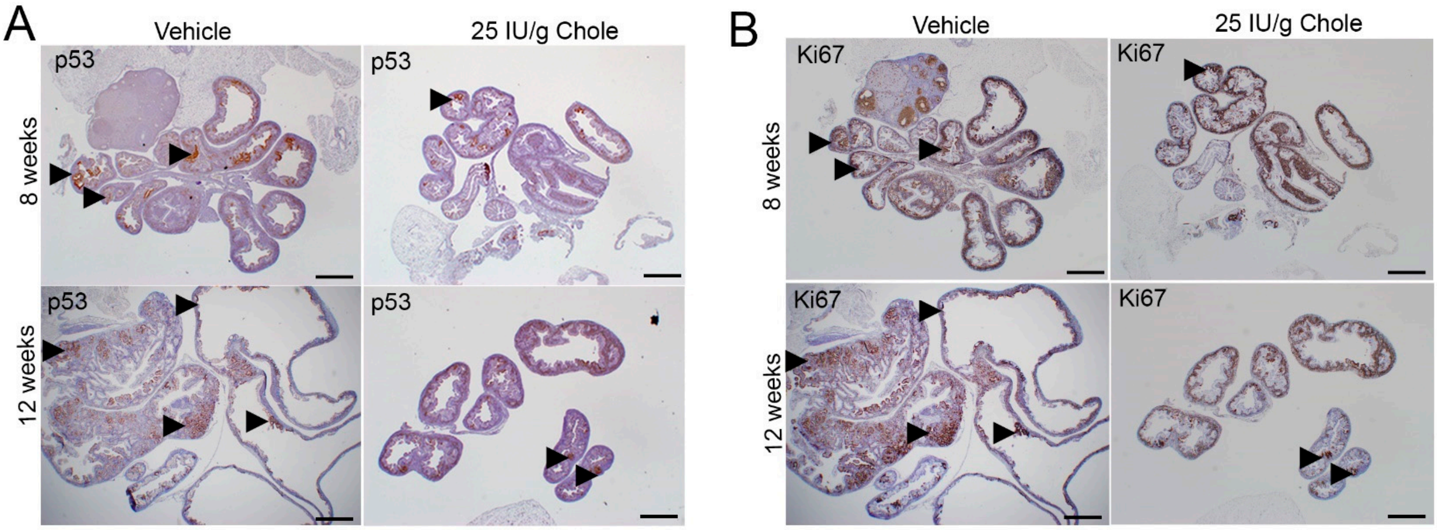

Figure S2. p53 and Ki67 positivity in the FT at 8 and 12 weeks of age in the feed group. A. Representative IHC of p53 following treatment with vehicle and Chole at 8 and 12 weeks. Images were captured at 2x magnification. Scale bar: 1mm. **B**. Representative serial IHC sections to images in (**A**) of Ki67 following treatment with vehicle and Chole at 8 and 12 weeks. Images were captured at 2x magnification. Scale bar: 1mm. Triangle highlights fallopian tube regions where p53 positive cells overlap with Ki67 positive cells in (**A**).

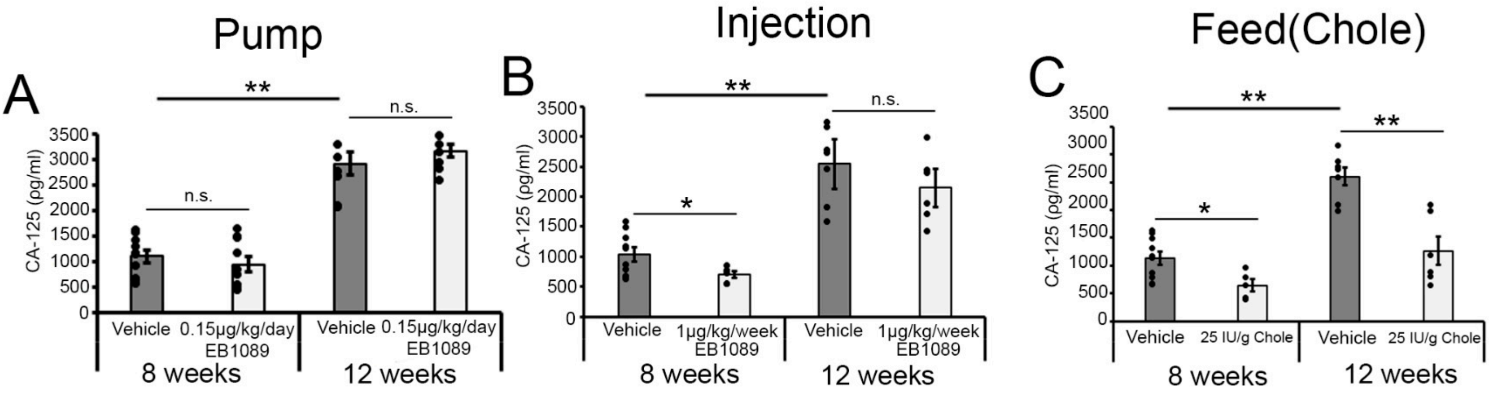

Figure S3. CA-125 measurements in vehicle and vitamin D group at 8 and 12 weeks of age. A. CA-125 levels in the plasma of the pump group at 8 weeks, vehicle (n=10) and EB1089 (n=10) and at 12 weeks, vehicle (n=8) and EB1089 (n=8). **B**. CA-125 levels in the plasma of the injection group at 8 weeks, vehicle (n=10) and EB1089 (n=6) and at 12 weeks, vehicle (n=7) and EB1089 (n=6). **C**. CA-125 levels in the plasma of the feed group at 8 weeks, vehicle (n=10) and Chole (n=5) and at 12 weeks, vehicle (n=7) and Chole (n=6). CA-125 measurements is reported in ρg/ml, mean ± SEM, *(p<0.05), **(p<0.005), T-test.

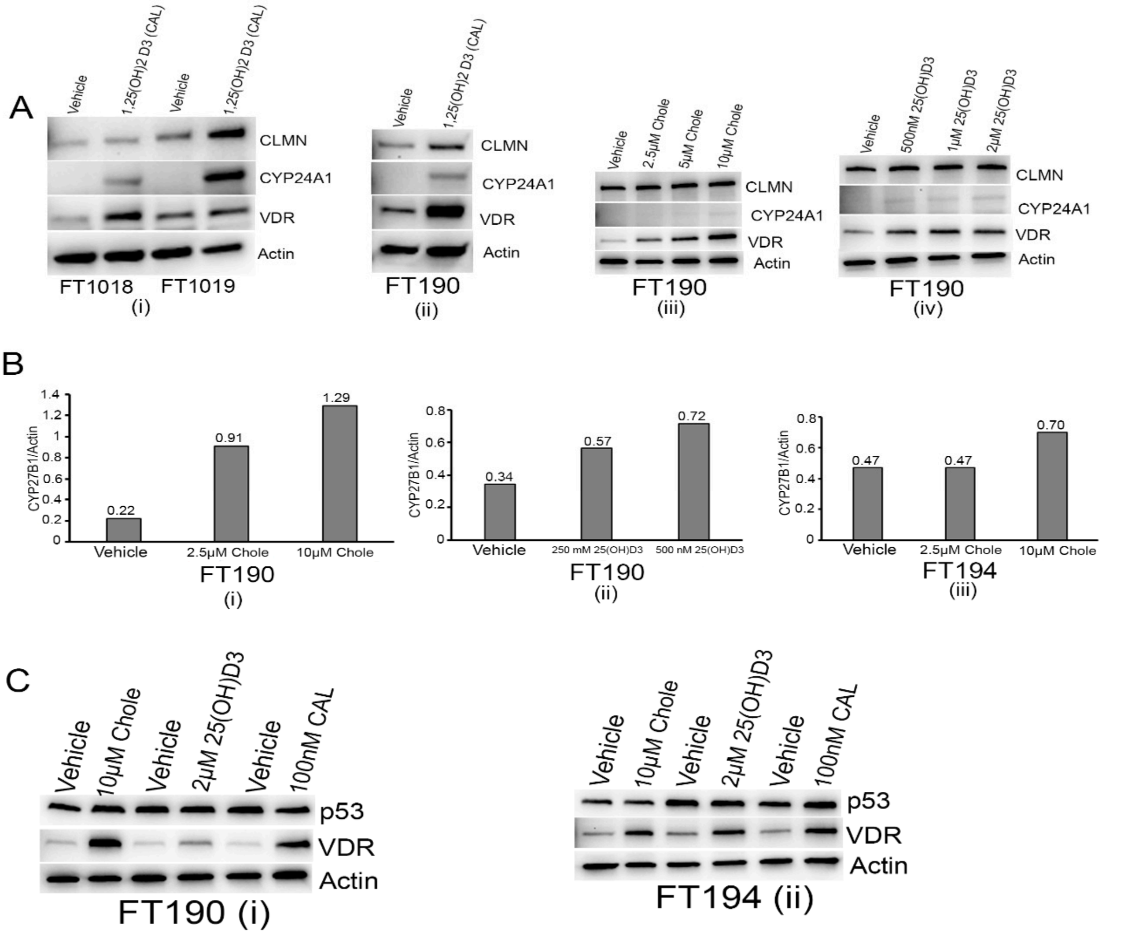

Figure S4. Cholecalciferol activates vitamin D-responsive genes in vitro, but does not alter p53 expression. A. Induction of Calmin (CLMN), CYP24A1 and VDR protein by 1, 25(OH)2 D3 (CAL) in primary FTE cells (i)(FT1018 and FT1019) and (ii) FT190 (expressing inactivated p53). Induction of CLMN, CYP24A1 and VDR protein by Chole (iii) and 25 (OH)D3 (iv) in FT190. B. CYP27B1 protein expression following treatments with Chole (i) and 25(OH)D3 (ii) in FT190 and Chole (iii) in FT194. CYP27B1 was normalized to actin. The values above each bar on the graph represents the CYP27B1: Actin ratio. C. Western blot of p53 following treatment with Chole, 25(OH)D3 and CAL in FT190 and FT194. VDR was used as a positive control for vitamin D responsiveness in the cells. Actin was used as a loading control.

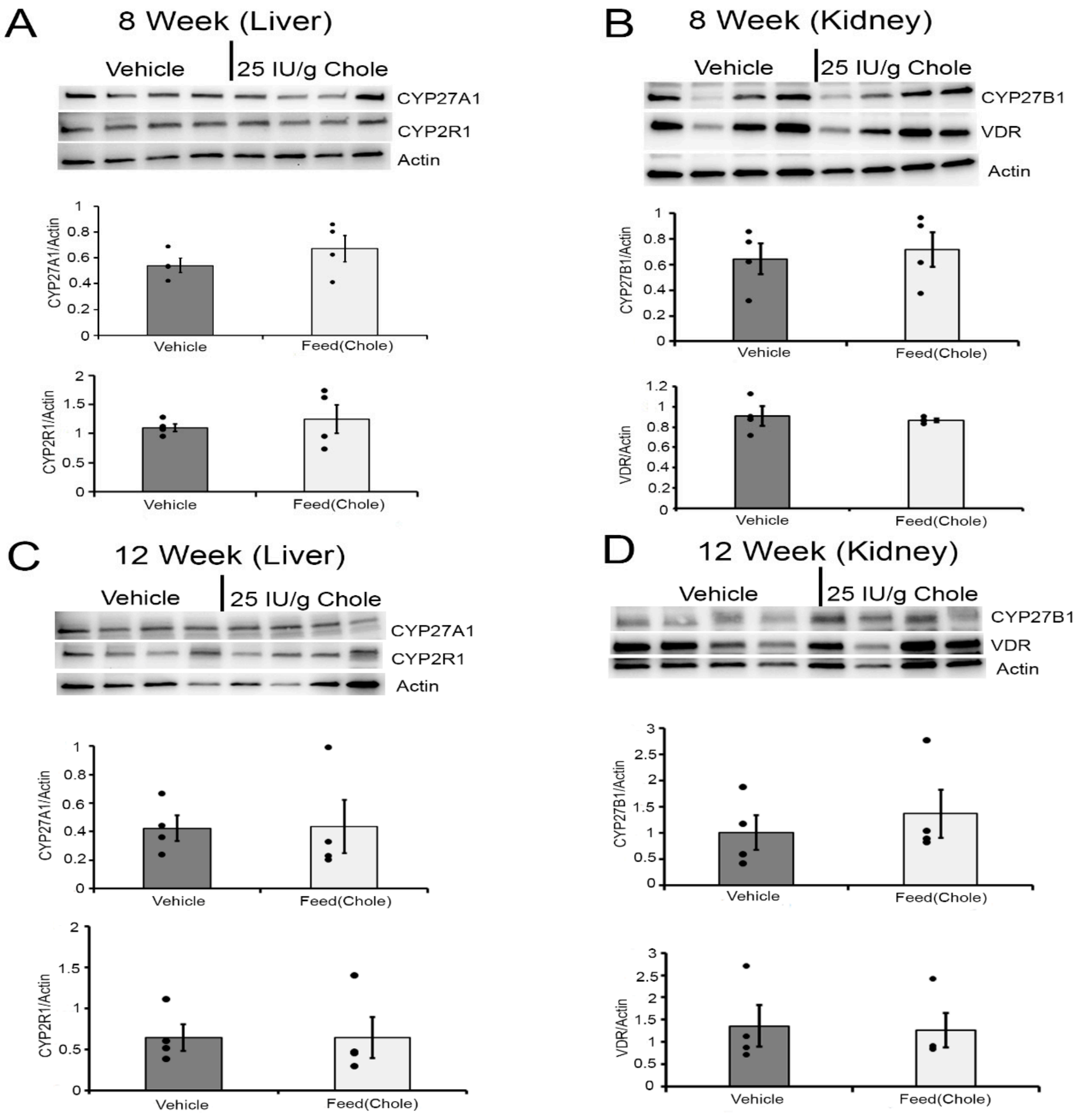

Figure S5. Protein expression of cytochrome P450 enzymes involved in the conversion of cholecalciferol to active vitamin D in the liver and kidney in the feed trial. A. Western blot of CYP2R1 and CYP27A1 in the liver of 8-week-old mice from the vehicle (n=4) and feed (n=4) group. **B**. Western blot of CYP27B1 and VDR in the kidney of 8-week-old mice from the vehicle (n=4) and feed (n=4) group. **C**. Western blot of CYP2R1 and CYP27A1 in the liver of 12-week-old mice from the vehicle (n=4) and feed (n=4) group. **D**. Western blot of CYP27B1 and VDR in the kidney of 12-week-old mice from the vehicle and feed group. For densitometry calculations, protein expression was normalized to actin. mean ± SEM, T-test.

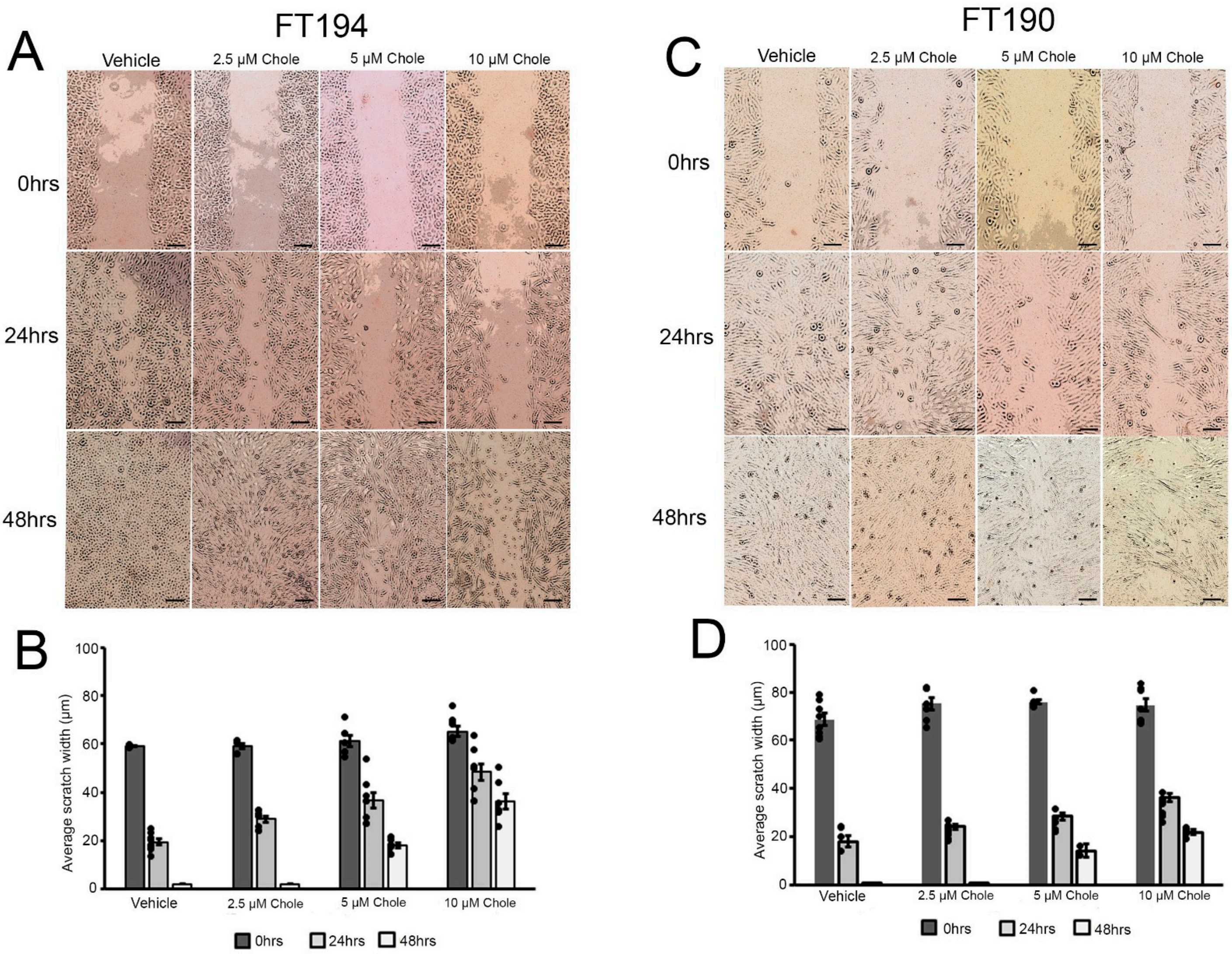

Figure S6. Effect of cholecalciferol on scratch/wound healing assay. A. FT194 cell culture treated with vehicle and different doses of Chole at 0, 24 and 48hrs. **B**. Quantification of the scratch width in FT194 at 0, 24 and 48hrs **C**. FT190 cell culture treated with vehicle and different doses of Chole at 0, 24 and 48hrs. **D**. Quantification of the scratch width in FT190 at 0, 24 and 48hrs. 4-7 measurements were obtained to calculate the average scratch width at each time point. Scale bar: 20μm. mean ± SEM
